# Epitaph Prof. Dr. med. Christof Jürg Daetwyler: *8.8.1964 – †11.12.2020

**DOI:** 10.3205/zma001431

**Published:** 2021-02-15

**Authors:** Sissel Guttormsen, Kai P. Schnabel, Wolf Langewitz

**Affiliations:** 1 Universität Bern, Institut für Medizinische Lehre, Bern, Switzerland; 2 Universität Bern, Institut für Medizinische Lehre, Abteilung für Unterricht und Medien, Bern, Switzerland; 3 University Hospital Basel, Psychosomatik – Kommunikation, Basel, Switzerland

## Epitaph

We received the unexpected message about Christof Daetwyler’s sudden death with great sadness. He passed away in the morning of December 11^th^ at his home in Pirenopolis, Gois, Brasilia. Christof Daetwyler was born in Zollikerberg in Zürich, Switzerland, he was the older of two brothers. A significant person we have worked with and greatly appreciated since very many years suddenly slipped out of our lives, leaving a great and irreplaceable loss. 

Christof was well known and highly esteemed for his contributions to medical education and his innovative technological approaches, many of which were pathbreaking. For many members of the Association for Medical Education (GMA) [https://www.gma-dach.org], as co-organiser of the Slice of Life community and in the AMEE, he was a friend, a colleague and an expert who regularly shared his new ideas at these conferences. 

His success was built on a broad multi-professional education, on talent and insistency: 

He completed his basic education in Zürich. His thirst for thorough knowledge in the fields that interested him, first led him to the “Higher School of Fine Arts” in Zürich, where he completed classes in painting and drawing (1985-1986). After completion of the art studies, he studied medicine at the university of Zürich (1986-1993). With his background in the arts, it was clear to him that he wanted to use his medical skills outside the clinical field. Already during his medical education, he worked part time as a data-base expert in two different companies. Then, from 1994 he started to actively combine his medical and media-technical skills at the “Department for Instructional Media” (AUM), at the “Institute of education and further medical training” in Bern, Switzerland (IAWF, today “Institute for Medical Education”, IML [https://www.iml.unibe.ch]). At the same time, working part time at the AUM, he studied documentary movie making at the “Higher school of fine Arts” in Bern (1995-1996). Continuing to work full time at the AUM, he completed his MD thesis (1999) on the issue of technology enhanced medical education. During his time in the AUM, he contributed significantly to the development of computer assisted learning modules for medical education (e.g.: neurology/headache interactive). His work in this area was pioneering and he developed several price-winning interactive learning programs. In his own words, he described his time at the AUM as follows: 

*When I graduated from medical school Zurich in 1993, I did a short stint at the Swiss National Television Company in its public health education department. In 1994 I started my professional career with two jobs at the University of Bern Medical School simultaneously: As a curriculum designer for the 3rd year and as a producer of video and developer of (then very new) computer applications for medical education at the Department of Educational Media (AUM). After one year, I concentrated my energy on my job at the AUM. As often in life, it’s people who are the most important ingredient to success. In my case, I was extremely lucky to meet and collaborate with Marco Mumenthaler, a “Grand Senior” of Neurology – and a great teacher. Our work was very productive and successful, in terms of projects (they received many awards and were translated into different languages) as well as for personal reasons since we became friends. I stayed at the AUM for almost 8 years, until 2001 *[http://webcampus.drexelmed.edu/interactive/cda/cv/index.html].

In 2001, Christof set off to the United States to start a new chapter of his remarkable career. As a research assistant professor at the “Department of Community and Family Medicine”, at Dartmouth Medical School, he was invited by Joe Henderson to work as a lead designer and multimedia Developer at the interactive media laboratory (directed by Joe Henderson). After three creative years, he changed to the Drexel University College of Medicine in 2004 as a research assistant professor in the Department of Family, Community and Preventive Medicine (directed by Dennis Novack), where he started a successful and creative cooperation with Dennis Novack. Christof described his activities at Drexel as follows: 

*In October 2004, I joined the Faculty of Drexel University College of Medicine in Philadelphia as an assistant research professor. My task was to continue on my way and to research, develop and integrate computer technology for the enhancement of medical education. My first large-scale project was “doc.com”, a series of 41 media-rich on-line modules for the teaching, learning, and assessment of skills needed in healthcare communication. Another project I'm working on is “WebOSCE”, an on-line technology to allow students to practice new knowledge and skills on on-line standardized patient *[http://webcampus.drexelmed.edu/interactive/cda/cv/index.html]*.*

His productions use technological possibilities and the internet in a visionary way. He truly enabled digital transformation in medical teaching long before the current pandemic showed how important this is. Many of his productions, being successfully introduced in the US, also have become appreciated in the German speaking countries in various medical schools. He supported projects in Switzerland (e.g. Bern, Basel) and in Germany (e.g. LMU and TUM in Munich, Ulm, Mannheim). Since 2018 he also held a formal position at the University Basel, in the student dean’s office for the development of a web-based video processing application supporting students in reflecting on their own performance. Indeed, the “doc.com” [https://webcampus.drexelmed.edu/doccom/user/] as well as the “Web-OSCE” [http://www.iamse.org/websem/webosce-an-online-tool-for-remote-encounters-between-learners-and-standardized-patients-for-the-practice-assessment-and-remediation-of-clinical-skills/] projects represent typical achievements of Christof’s work and add to the series of fruitful and stimulating cooperation with colleagues, this time with Dennis Novack. Today “Doc.Com” is successfully implemented as a learning resource for doctor patient communication in many prestigious universities in the US and abroad. An offspring of Christof’s work is already well established in the German speaking countries in Europe. With the support of Dennis Novack, he initiated the generation of a European, German speaking, version of the “Doc.Com” platform, simply called “DocCom.Deutsch” [https://doccom.iml.unibe.ch/startseite/]. The first release was a team effort, with Christof, Wolf Langewitz (medical faculty Basel), Kai Schnabel and Sissel Guttormsen (both IML), supported from the Novartis foundation for men and environment and by many experienced experts from the GMA community (among others: Götz Fabry, Martin Fischer, Ueli Grüninger, André Karger, Claudia Kiessling, Dunja Nicca, Ulrich Schwantes, Michaela Wagner-Menghin). As for the US-original, the German production was created based on the insight that successful doctor-patient communication can be trained, and that online media, when designed well, deliver important resources for basic communication training. This tool, today managed and developed further by the IML, somehow bridges Christof’s early work at the IML with his later achievements. It was important for Christof to close this circle, and we are grateful for the enrichment this cooperation has become to us. It was also an important concern for him to support advancements in the education and particularly the employment of simulated patients (SPs). He promoted their education and enabled them to work from home, organising their encounters flexibly directly with the respective students (with Web-Encounter [1]). With this tool, SPs in USA are engaged as experts training medical students in various communication skills, giving structured feedback about their performance. 

Christof strength was to inform and to bring out the message about the importance and relevance of high-quality media productions for the sake of effective medical education. His publications also include, book chapters and editorship for four books, a long list of about 70 communications (accessible online [http://webcampus.drexelmed.edu/interactive/cda/publications/index.html]), as well as conference talks and workshops. As an engaged presenter, he was regularly invited as a speaker at national and international events. Throughout his career, he also continued to use his talent and expertise in arts e.g. as a photographer and as a painter. 

In 2019 Christof decided to retire early from Drexel. He moved with his wife Magalene Daetwyler-Pina to Brasilia. There he continued to work as a freelancer, as a researcher and developer, he helped is step-son set up a brewery, and started organising music festivals. 

We will miss his enthusiasm, never ending energy for new ventures, creative ideas - and above all his heartly and generous nature; he was a remarkable person, self-assure and vulnerable at the same time. Christoph saw his success as a medical educator and media specialist in that he was trained in different disciplines, and thereby could combine his knowledge in the arts, video-documentation and medicine for the development of complex learning applications. He was an idealist tirelessly taking on a great workload and with a strong commitment to get his projects done. He managed successfully the art of promoting his ideas and to nourish recognition for his remarkable work. His enthusiasm was sometimes a curse and a blessing at the same time, particularly when the potential of his ideas could not be realised. We are left with gratitude for what he has created and a great loss for the new work that will no longer emerge. 

## Competing interests

The authors declare that they have no competing interests. 
